# Current‐Driven Nonreciprocal Response of Nonequilibrium Skyrmions

**DOI:** 10.1002/advs.202524224

**Published:** 2026-03-20

**Authors:** Xiuzhen Yu, Soju Furuta, Xichao Zhang, Yi‐Ling Chiew, Kiyomi Nakajima, Kosuke Karube, Naoya Kanazawa, Yasujiro Taguchi, Yoshinori Tokura, Masahito Mochizuki, Fumitaka Kagawa

**Affiliations:** ^1^ RIKEN Center for Emergent Matter Science Wako Japan; ^2^ School of Science The Institute of Science Tokyo Tokyo Japan; ^3^ Department of Applied Physics Waseda University Tokyo Japan; ^4^ Institute of Science Tokyo The University of Tokyo Tokyo Japan; ^5^ RIKEN Baton Zone Program TRIP Headquarters Wako Japan; ^6^ Tokyo College The University of Tokyo Tokyo Japan

**Keywords:** current crowding effect, in situ Lorentz TEM, magnetic skyrmion, micromagnetic simulation, nonreciprocity

## Abstract

Electrical control of spins and their collective textures, such as topologically protected skyrmions, is highly desirable for unlocking their potential in future electronic technologies. Here, we design and fabricate thin films of FeGe and Co_8_._5_Zn_8_._5_Mn_3_, each containing a small central hole comparable in size to individual skyrmions, to investigate how nonuniform electron flow influences skyrmions and drives nonreciprocal skyrmion dynamics. Using in situ real‐space imaging, we observe that current crowding around the hole triggers skyrmion nucleation and produces direction‐dependent skyrmion motion, in agreement with theoretical models and numerical simulations. Notably, the current‐driven nonequilibrium skyrmion reveals a striking manifestation of topological spin textures, offering new opportunities for information processing that leverages skyrmion nonreciprocity, such as in reservoir computing.

## Graphical abstract

A skyrmion holds great promise for spintronic applications owing to its intrinsic topological stability and high mobility under ultra‐low current excitation. Here, by means of in situ electron microscopy, we demonstrate a skyrmion diode effect and the associated current‐driven nonreciprocal skyrmion dynamics in a designed FeGe‐based microdevice. These results open new avenues for exploiting skyrmions in future low‐power spintronic engineering.

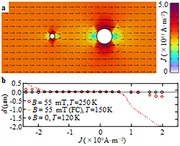

## 1 Introduction

Electrical control of spins is essential to nonvolatile memory devices [1, 2, 3], neuromorphic computing [4], and energy‐efficient logic operations [5, 6], where data are stored and processed using electron spins rather than electron charges. The ability to electrically manipulate topological spin textures, such as magnetic skyrmions, holds great promise for the development of low‐power electronic devices [7, 8, 9, 10, 11]. Nanometric skyrmions emerge in magnets with the intrinsic Dzyaloshinskii–Moriya interaction (DMI) [12], a consequence of their non‐centrosymmetric crystal structure [7, 13, 14, 15, 16]. They can also form in engineered ferromagnetic/antiferromagnetic heterostructures with spin‐orbit interactions when subjected to external fields [17, 18]. The potential applications of skyrmions extend to spin‐based electronic devices and neuromorphic computing [19, 20, 21, 22, 23]. In the former, controlled in‐line motion of skyrmions is vital for racetrack memory devices [10], while in the latter, nonreciprocal motion of skyrmions could enable novel logic operations [22, 23]. The nonreciprocal response arising from topological spin textures and intrinsic material properties has been predicted in skyrmion‐hosting MnSi, FeGe, and Co_8_._5_Zn_8_._5_Mn_3_ [16], and confirmed experimentally in MnSi, where nonreciprocal transport phenomena of skyrmion strings emerge under AC current excitation [24]. Specifically, distortions in skyrmion strings generate an emergent electromagnetic field, leading to a nonlinear Hall effect dependent on current direction.

When an electric current flows through skyrmions, spin‐polarized electrons interact with localized spins via spin‐transfer torque (STT) [7, 24, 25], inducing effects such as skyrmion translation and Hall motion [7]. While uniform current‐driven skyrmion dynamics via STT has been extensively studied [25, 26, 27, 28, 29], the interplay between nonuniform electric currents and skyrmion dynamics is an emerging topic in condensed matter physics and spintronics. The research explores how spatially varying electric currents influence skyrmion nonreciprocity [30, 31, 32]. Beyond driving skyrmion motion, nonuniform currents facilitate skyrmion nucleation, skyrmion deformation [33, 34, 35, 36] or disordered skyrmions [37], and impart unique, direction‐dependent dynamics to magnetic structures [38]. From such a fundamental magnetism perspective, understanding how current crowding assists in the formation of topological spin textures could unveil new pathways for controlling electron spins.

Theoretical studies have predicted that nonuniform‐current‐driven skyrmion dynamics arise in confined geometries such as narrow channels and notched thin films, where skyrmion–boundary interactions and hydrodynamic electron flow play crucial roles [39]. Confinement strongly affects skyrmion generation and motion, producing steady‐state velocities that depend on both the current density and the skyrmion's proximity to edges—behavior reminiscent of domain‐wall dynamics in ferromagnets [32]. Beyond geometric constriction, introducing a central hole into a thin film can also generate nonuniform electron flow and current crowding [40]. Around such a hole, current crowding significantly impacts skyrmion nucleation and dynamics. This nonuniform current distribution locally modifies the spin torques in helimagnets with intrinsic DMI, giving rise to asymmetric (nonreciprocal) skyrmion motion under applied current. In addition to the nonreciprocity of skyrmion, the skyrmion‐hole interaction can be used to manipulate the collective flow behavior of skyrmions [41].

To demonstrate the nonreciprocal dynamics of nonequilibrium skyrmions under nonuniform current excitations, we perform in situ Lorentz transmission electron microscopy (TEM). This technique enables directly visualizing topological spin textures and their dynamics with spatial resolution below 2 nm and temporal resolution on the order of 5 ms [42, 43]. Compared with transport‐based measurements of skyrmion nonreciprocity, real‐space imaging provides deeper insight into the underlying dynamics under various conditions. In particular, the high spatial and temporal resolution of Lorentz TEM allows us to unambiguously resolve skyrmion motion and distinguish it from other phenomena, such as domain‐wall dynamics, thereby offering a more comprehensive understanding of nonreciprocal skyrmion behavior. In this work, we engineer thin chiral magnetic films of FeGe and Co_8_._5_Zn_8_._5_Mn_3_ incorporating nanoscale holes with dimensions comparable to individual skyrmions. This device geometry enables controlled current inhomogeneity while minimizing structural damage to the sample. By intentionally introducing asymmetric holes along the current path, we break structural symmetry and enhance device nonreciprocity. Using real‐space in situ Lorentz TEM combined with micromagnetic simulations, we directly visualize the asymmetric nucleation and nonreciprocal motion of skyrmions under nonuniform electron flow, revealing pronounced nonreciprocal skyrmion dynamics induced by current crowding. These results provide direct experimental evidence clarifying the skyrmion diode effect in a realistically engineered microdevice platform and establish current crowding as an effective strategy for controlling topological spin textures.

## 2 Results and Discussion

### 2.1 Skyrmion Formation Under Nonuniform Current Excitation

**FIGURE 1:**
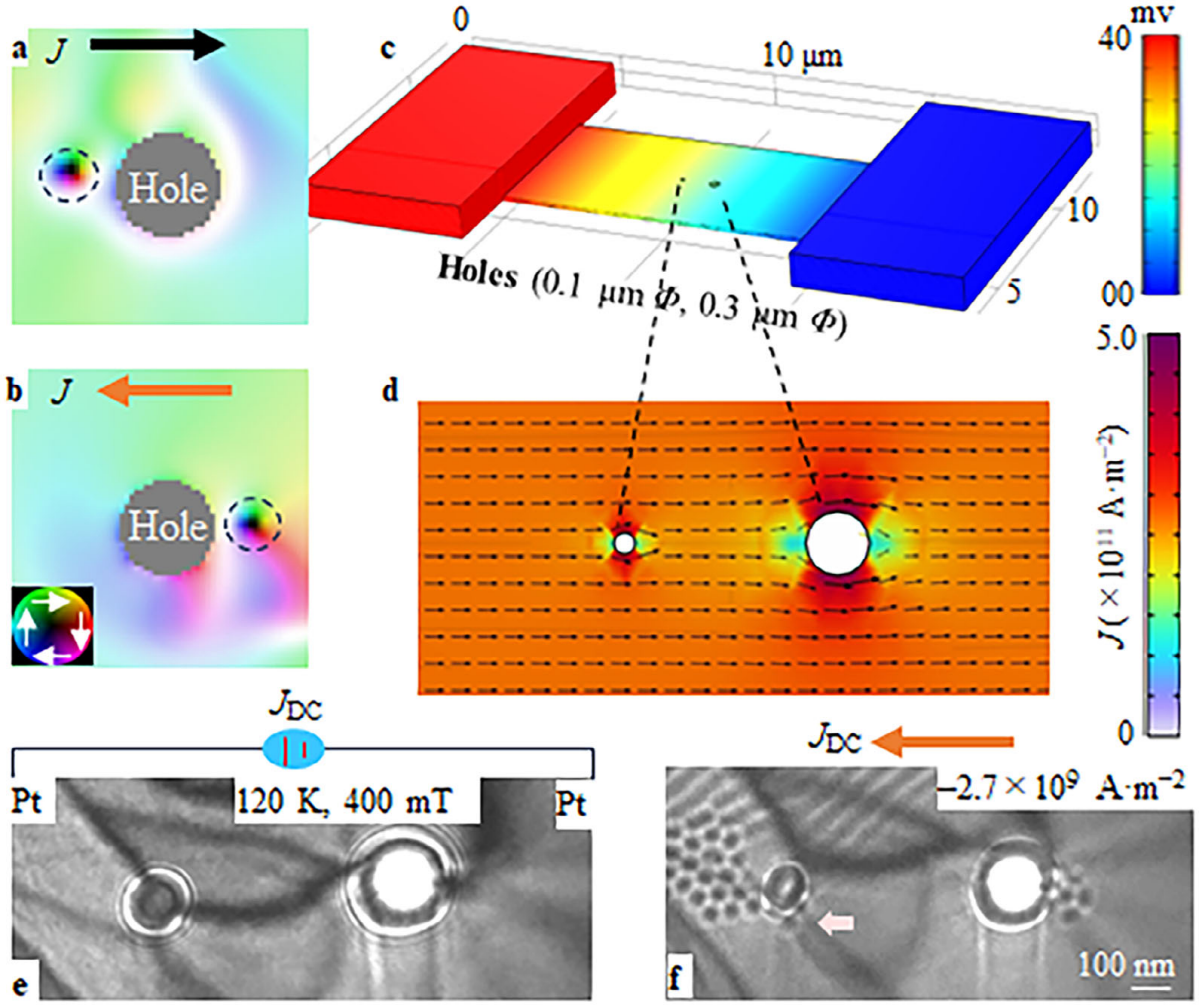
Skyrmion formations under nonuniform current flows in a thin chiral‐lattice magnet FeGe with two designed holes (0.1 and 0.3 µm in diameter). (a,b) Snapshots from micromagnetic simulations showing skyrmion formations around the 0.1‐µm hole under transverse current flow: (a) current flowing from left to right in FeGe; (b) current flowing from right to left. Dashed black lines mark individual skyrmions. The color wheel in the inset of Figure 1b indicates the in‐plane magnetization directions of spin textures. (c,d) The voltage (c) and current density (d) maps, calculated using multiphysics software COMSOL based on a finite element model [44], illustrating a nonuniform current distribution around the holes (d). Colors and arrows represent the magnitude and direction of voltage and current, respectively. (e,f) Lorentz transmission electron microscopy (TEM) images taken under a 400 mT normal field at a constant temperature of 120 K: (e) without current flow and (f) after current flow at *J* = −2.7 × 10^9^ A·m^−^^2^ for 100 ms, with the current flowing from right to left.

First, we investigated skyrmion dynamics in a FeGe micromagnet model system under uniform and nonuniform current flows using micromagnetic simulations based on the Landau‐Lifshitz‐Gilbert (LLG) equation (see details in Methods section). The results indicate that the threshold for generating skyrmions from nontopological states, such as conical and ferromagnetic states, is lower under nonuniform currents than under uniform currents (Figure S1). Magnetization dynamics in the FeGe micromagnet with a central hole clearly demonstrate the skyrmion nucleation around the hole (Movie S1), whereas no skyrmions appear when a uniform electric current of the same magnitude flows through a flat FeGe (Movie S2). Notably, the hole size (∼100 nm) is comparable to the skyrmion diameter (∼80 nm) in FeGe. The simulations suggest that the hole plays a crucial role in the electrical manipulation of skyrmions in FeGe. Figure 1a,b, has demonstrated that a single skyrmion initially forms on the left side of the 100‐nm hole (Figure 1a) when a current exceeding 5 × 10^11^ A·m^−^^2^ flows through the FeGe. Conversely, when the current is reversed and surpasses approximately −5 × 10^11^ A·m^−^^2^, the skyrmion appears on the right side of the hole (Figure 1b). The formation of a skyrmion occurs within a few nanoseconds (Movies S3 and S4). With increasing the elapsed time of DC‐current flow, subsequent skyrmion formations occur, and skyrmions move approximately against the current direction. When the current flows from left to right, the skyrmions exhibit translational motion along the upper edge of the hole (Movie S3), whereas they move along the lower edge when the current direction is reversed (Movie S4), indicating dynamically asymmetric skyrmion formations triggered by current crowding. This asymmetry may arise from the nonuniform current distribution, which locally modifies the STT that facilitates skyrmion generation.

To experimentally confirm the magnetization dynamics driven by nonuniform currents and the subsequent electrical creation of skyrmions, two holes with 100 and 300 nm in diameter were fabricated in a 100‐nm‐thick FeGe plate with in‐plane dimensions of 10 µm in width and 15 µm in length. The plate is connected to two Pt electrodes at its ends, generating a potential (voltage) distribution when a current flows through it, as shown in Figure 1c. The potential distribution was calculated precisely using a finite element method simulation [44]. The corresponding magnified current density map around the holes, as shown in Figure 1d, demonstrates that the current crowding around the hole forms high‐density regions at the hole edges and low‐density regions further away from the hole.

**FIGURE 2:**
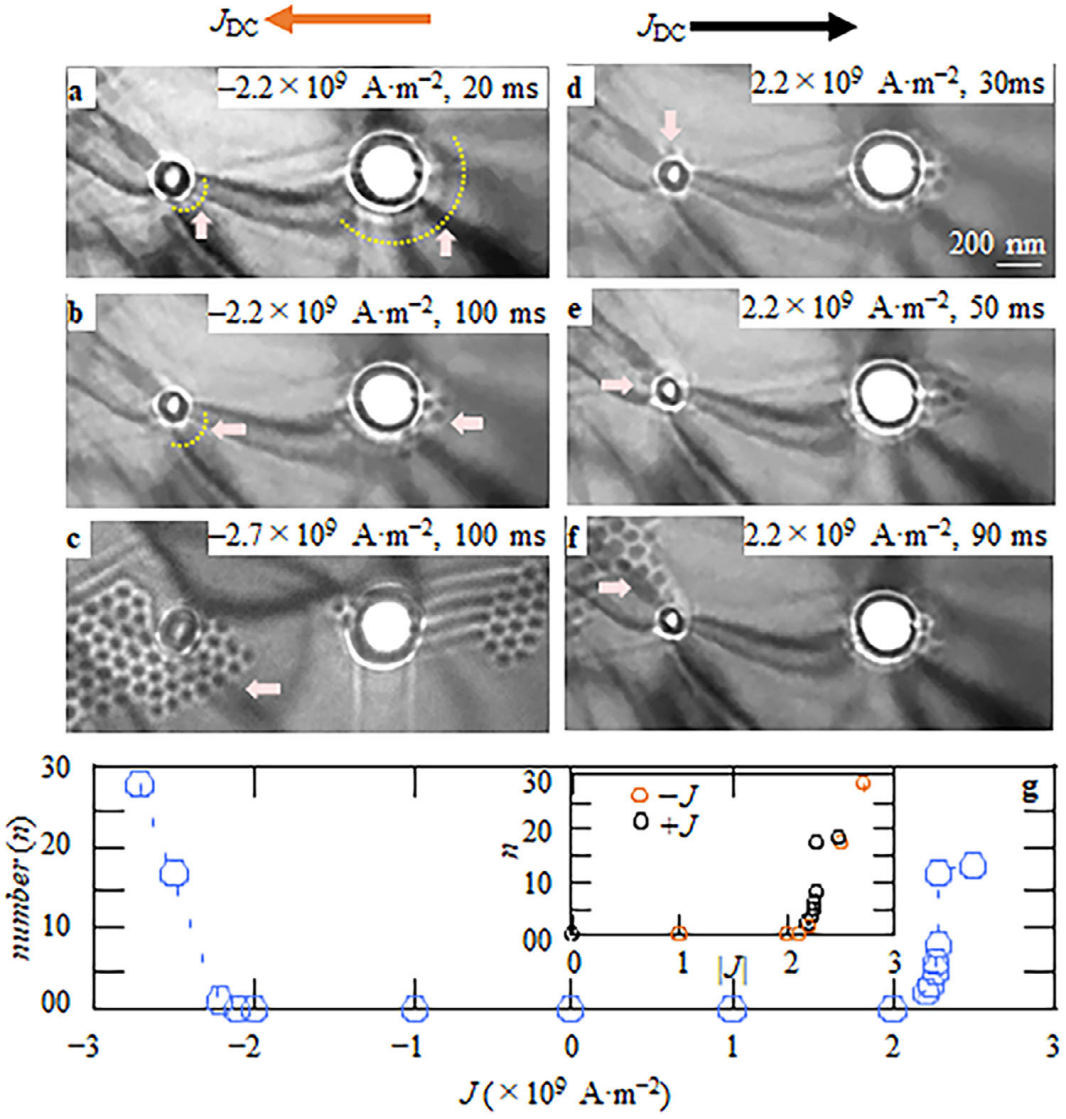
Nonreciprocal dynamics of skyrmion nucleation under current crowding. (a–c) Lorentz TEM images captured under “negative” current flow (right to left). (a,b) Images taken at a constant current of *J* = −2.2 × 10^9^ A·m^−^^2^ with varying elapsed time. (b,c) Images captured at an elapsed time of 100 ms with increasing current strength. (d–f) Lorentz TEM images recorded under “positive” current flow (left to right) with varying elapsed time while maintaining a constant current of 2.2 × 10^9^ A·m^−^^2^. (g) The number of skyrmions (*n*) generated at different electric currents, measured at a fixed elapsed time of 50 ms. The inset shows a plot of the number of skyrmions vs. the current strength.

An initial conical state has been prepared as a monotonic contrast in a Lorentz TEM image, as shown in Figure 1e. The asymmetric skyrmion formations around the hole under nonuniform current flow have been demonstrated experimentally in a Lorentz TEM image observed after applying a current of −2.7 × 10^9^ A·m^−^^2^ (Figure 1f). The detailed procedure of in situ Lorentz TEM observations of current‐induced skyrmion formation is as follows: First, a DC current is gradually increased from 0 to ±3 × 10^9^ A·m^−^^2^ through the FeGe to determine the threshold current density for skyrmion generation, found to be ±2.2 × 10^9^ A·m^−^^2^. Next, a current density of −2.7 × 10^9^ A·m^−^^2^ is maintained through the FeGe for 100 ms, after which a Lorentz snapshot is recorded, as shown in Figure 1f. An in situ Lorentz TEM movie (Movie S5) reveals that skyrmions initially form on the right side of both holes and then appear to rotate around them under a current of −2.2 × 10^9^ A·m^−^^2^, the threshold for skyrmion creation. When the current is reversed, skyrmions preferentially form at the top left of the 100‐nm hole (Movie S6). Due to the millisecond‐scale frame rate of experimental observation—six orders of magnitude longer than the simulations—it is challenging to directly reproduce the micromagnetic simulations shown in Movies S3 and S4. Experimentally, a skyrmion cluster accompanied by in‐plane helices emerges around the 100‐nm hole at an elapsed time of 100 ms (Figure 1f), contrasting with the single conical domain state (lacking in‐plane contrast) in Figure 1e. Thus, the electrical manipulation of magnetic skyrmions in a conical state has been demonstrated within a thin helimagnet FeGe under nonuniform current flow. Detailed evidence of electrically manipulated magnetic skyrmions is presented in Figure 2, as follows.

### 2.2 Nonreciprocal Nucleation Behavior of Skyrmions Induced by Current Crowding

Sequential Lorentz TEM images observed at different elapsed‐time points under DC current flows are shown in Figure 2. The state is initialized to the conical state before each current flows through the sample, and it is also offset when changing the current's strength and direction. When a threshold current of –2.2 × 10^9^ A·m^−^^2^ passes through a conical state with an out‐of‐plane wavevector (Figure 1e), arc‐shaped magnetic contrasts corresponding to in‐plane spin textures (highlighted by yellow dashed lines) appear at the lower‐right side of both holes at an elapsed time of 20 ms (Figure 2a). This suggests that the current partially causes the wavevector's reorientation from the out‐of‐plane into the plane, forming in‐plane helices around the holes. As the elapsed time of current flow increases (Figure 2b) or the current rises to –2.7 × 10^9^ A·m^−^^2^ (Figure 2c), the in‐plane spin textures evolve into skyrmions. Subsequently, skyrmions crystallize at the lower side of the 100‐nm hole, while a skyrmion cluster moves away from the 300‐nm hole. The movement of the skyrmion cluster, accompanied by the extension of in‐plane helices in the direction opposite to the current flow (Figure 2c), phenomenologically suggests a repulsive force acting on the skyrmion cluster from the edge of the 300‐nm hole.

In line with the micromagnetic simulations shown in Movies S3 and S4, skyrmions begin to form and migrate to the upper side of the hole under a “positive” current flow (from left to right across the plate), as indicated by the shorter arrows in Figure 2d,e. Further increasing the elapsed time of current flow at a constant current strength allows skyrmions to accumulate, leading to the formation of a skyrmion cluster or a lattice at the upper side of the 100‐nm hole (Figure 2f). When the current direction is reversed, the aggregated skyrmions exhibit a similar behavior, while those localized on the lower side of the 100‐nm hole (Figure 2c) persist, highlighting the directional influence of the current on skyrmion formation around the 100‐nm hole. This nonreciprocal skyrmion formation, driven by current crowding, has also been observed in another thin helimagnet Co_8.5_Zn_8.5_Mn_3_ with a single central hole (Figures S2 and S3).

Figure 2g displays the number of skyrmions generated in the FeGe plate measured at a constant time (50 ms) as a function of current intensity. The results reveal that below the threshold current of ∼ ±2.2 × 10^9^ A·m^−^^2^, skyrmions are not created in the FeGe plate. However, when the current approaches or exceeds this threshold, skyrmions are created by nonuniform electron flows, and the number of skyrmions increases with increasing current strength (Figure 2g). (To prevent device failure, a short elapsed time of 50 ms and relatively small current densities below ±3 × 10^9^ A·m^−^^2^ were employed.) Notably, the number of skyrmions differs depending on the current direction, due to the asymmetric pinning potential caused by the two holes with different sizes.

### 2.3 Current‐Driven Diode‐Like Response of Nonequilibrium Skyrmions

**FIGURE 3:**
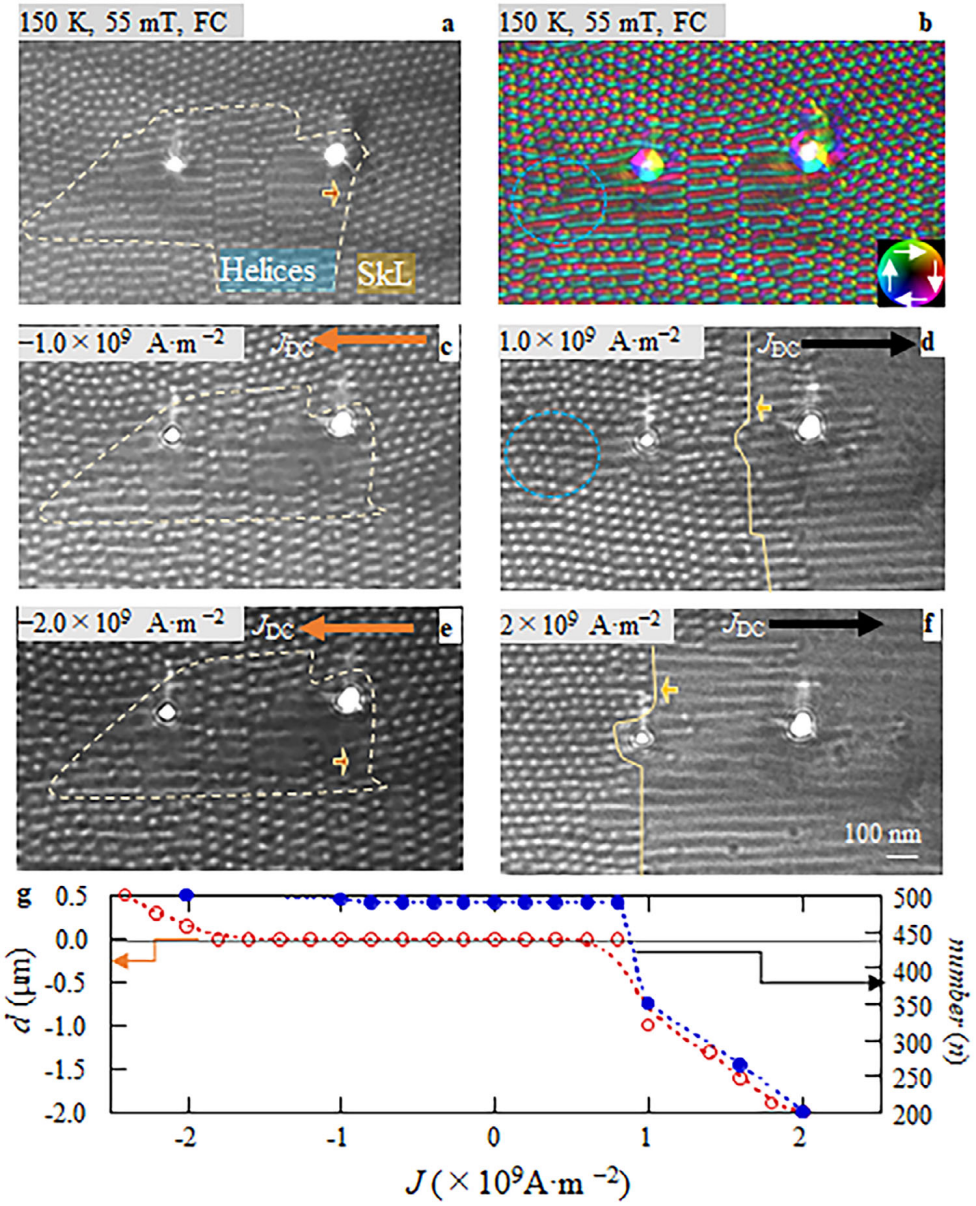
Diode effect of dynamical skyrmions under electric current flows at 150 K in FeGe with two differently sized holes. (a,b) Lorentz TEM configuration (a) and the corresponding field map (b) showing a hexagonal skyrmion lattice (SkL), along with heavily deformed skyrmions and in‐plane helices (circled with dashed lines) near the holes. The Lorentz TEM images were obtained at 150 K after field cooling (FC) from room temperature under a 55 mT field. The color wheel in the inset represents the in‐plane magnetization directions of spin textures. (c–f) Lorentz TEM images captured under “negative” (c,e) and “positive” (d,f) current flows, respectively. Solid lines indicate the boundary between the SkL and in‐plane helices. (g) The diode effect in current‐driven skyrmion dynamics, represented by the displacement (*d*) and the variation in the number of skyrmions (*n*) as a function of current (*J*).

The in situ Lorentz TEM observations of nonuniform current‐driven skyrmion dynamics reveal novel properties of spin textures, including wavevector alignment, deformations of thermodynamically stable skyrmions around holes (Figure S4), and a nonreciprocal response of non‐equilibrium skyrmions, as shown in Figure 3. Initially, a metastable skyrmion crystal (SkL) mixed with in‐plane helices (stripes around two holes) was created using a 55‐mT field cooling from RT to 150 K below the helimagnetic transition temperature (∼278 K, Figure S2) [16], as shown in a Lorentz TEM micrograph (Figure 3a) and the corresponding magnetic field map (Figure 3b). The temperature is maintained at 150 K to enhance the SkL metastability. When a current with a density of approximately 1 × 10^9^ A·m^−^^2^ flows from the right end to the left (the “negative” current) of the FeGe plate, both skyrmions and in‐plane helices remain nearly the same state (Figure 3c). In contrast, reversing the current direction (the “positive” current) induces skyrmion sliding motions against the current flow. This process is accompanied by the emergence of in‐plane helices on the right side of the plate and the transformation of elliptical skyrmions (outlined by blue dashed lines in Figure 3b) into circular skyrmions (circled by blue dashed lines in Figure 3d) on the left side of the plate (Figure 3d). Increasing the current to 2 × 10^9^ A·m^−^^2^ further amplifies the nonreciprocal properties of spin textures (see Figure 3e,f). As detailed in Figure 3g, when the current remains below ±1 × 10^9^ A·m^−^^2^, the spin textures, including metastable elliptical skyrmions, persist with minimal change. However, as the “positive” current exceeds 1 × 10^9^ A·m^−^^2^, skyrmions begin moving against the current flow, with the displacement (*d*) of the domain wall between skyrmions and in‐plane helices increasing. (For each current flow, the initial spin texture state was prepared as a mixture of metastable skyrmions and in‐plane helices, nearly the initial state shown in Figure 3a,b. All Lorentz TEM images were captured at an elapsed time of 100 ms.) Simultaneously, the number (*n*) of metastable skyrmions decreases significantly. In contrast, under the “negative” current flows, the *n* remains nearly constant, and the *d* is relatively small, even at current amplitudes exceeding 2 × 10^9^ A·m^−^^2^. The observed nonreciprocal phenomena in skyrmion dynamics under nonuniform current flow may be unique to the designed FeGe device with two holes of different sizes and the alignment along the current path, which act as asymmetric potential barriers for dynamical skyrmions, suggesting that skyrmions can overcome holes of comparable size but are blocked by larger holes, such as the 300‐nm‐diameter hole—three times the size of a skyrmion. Additionally, the emergence of in‐plane helices after skyrmion flow under the “positive” current is consistent with the thermodynamically stable helical state at lower temperatures and magnetic biases, as indicated by the FeGe phase diagram (Figure S2a). The nonreciprocal responses of spin textures in the helical and conical states at low temperatures (120 and 150 K), as well as in the skyrmion lattice state at higher temperatures (∼250 K), by tuning the bias magnetic field, have been further investigated. The diode effect is significantly more pronounced for metastable skyrmions generated by current in the helical or conical phases than for thermodynamically stable skyrmions formed near 250 K, close to *T*_*C* _ (Figure S5).

## 3 Conclusion

In conclusion, the in situ Lorentz TEM observations of metastable spin textures in thin helimagnet plates equipped with artificial holes have demonstrated the singular interactions between spin‐polarized electrons and local spins under nonuniform current flow. These interactions give rise to nonreciprocal skyrmion nucleation dynamics and diode‐like behavior of nonequilibrium skyrmions driven by current crowding, underscoring the potential of nanometric spin textures for future electronic devices.

## 4 Methods

### 4.1 Sample Preparations

Single crystals of FeGe and Co_8.5_Zn_8.5_Mn_3_ were grown using chemical vapor transport and the Bridgman method [45, 46], respectively. The space groups of both single crystals were determined through powder x‐ray diffraction and selected‐area electron diffraction measurements, identified as *P*2_1_3 for FeGe and *P*4_1_32*/P*4_3_32 for Co_8.5_Zn_8.5_Mn_3_, confirming their non‐centrosymmetric crystal structures [16]. Plates of FeGe and Co_8.5_Zn_8.5_Mn_3_ containing holes were prepared by thinning bulk samples with a dual‐beam system (Helios 5UX, Thermo Fisher Scientific). The thinning process was carried out at 30 kV using a Ga‐ion beam. To remove the damaged surface layer, the thin plates were then gently polished at 2 kV with a Ga‐ion beam. Finally, holes of varying sizes were created via Ga‐ion beam etching at 2 kV.

### 4.2 In Situ Lorentz TEM Observations

In situ Lorentz TEM was used to study dynamic spin textures under current flows, magnetic biases, and temperature control [42, 43]. Lorentz TEM is a specialized imaging mode of a transmission electron microscope that enables the visualization of magnetic configurations by exploiting the interaction of the electron beam with the magnetic fields within the sample. The Lorentz force caused by the magnetic field inside the material deflects the electron beam, thereby generating contrast in the Lorentz TEM images and revealing the spin textures in magnetic materials. In this study, we used a defocused Lorentz TEM mode, known as the Fresnel mode, to directly image the dynamics of spin textures under electric current flows. All Lorentz TEM observations here were performed using a transmission electron microscope (JEM‐2800, JEOL). A specialized cryogenic TEM sample holder with electrical terminals (HC3500, Gatan) was employed to apply a current bias to FeGe or Co_8.5_Zn_8.5_Mn_3_. The sample holder was powered by a source measure unit (Keithley 2601).

### 4.3 Micromagnetic Simulations

The magnetization dynamics are simulated using the commercial micromagnetic simulation package MuMax3 [47], based on the Landau‐Lifshitz‐Gilbert (LLG) equation augmented with adiabatic and non‐adiabatic spin‐transfer torque (STT), which is expressed as

(1) $$\begin{array}{ccc} \partial_{t}m & = & -\gamma_{0}m\times h_{\mathrm{eff}}+\alpha \left(m\times \partial_{t}m\right)+u\left(m\times \partial_{x}m\times m\right)\\ & & -\beta u\left(m\times \partial_{x}m\right), \end{array}$$

where ***m*** is the reduced magnetization, *t* is the time, γ_0_ is the absolute gyromagnetic ratio, and α is the Gilbert damping parameter. The adiabatic STT coefficient *u*  = |(γ_0_ℏ/μ_0_ *e*)|  · (*JP*/2*M*_S_), and β is the strength of the non‐adiabatic STT. The μ_0_ and *M*_S_ denote the vacuum permeability constant and the saturation magnetization, respectively. ℏ, *e*, *J*, *P* are the reduced Planck constant, the electron charge, the current density, and the spin polarization rate, respectively. The effective field $h_{\mathrm{eff}}=-\frac{1}{\mu_{0}M_{S}}\cdot \frac{\delta \varepsilon }{\delta m}$, where ε denotes micromagnetic energy density, including the ferromagnetic exchange interaction *A*, Dzyaloshinskii‐Moriya interaction *D*, and demagnetization *B*_d_, and applied magnetic field *B*_e_ terms, described as

(2) $$\begin{array}{c} \varepsilon =A{\left(\nabla m\right)}^{2}+D\left[m\cdot \left(\nabla \times m\right)\right]-\frac{M_{S}}{2}\left(m\cdot B_{d}\right)-M_{S}\left(m\cdot B_{e}\right). \end{array}$$

The default magnetic parameters are: γ_0_ =  2.211 × 10^5^ m A^−1^ S^−1^, α  =  0.03 *M*_S_ =  384 kA m^−1^, *A*  =  4.75 pJ m^−1^, and *D*  =  0.853 mJ m^−2^. For the sake of simplicity, we assume that *P*  =  1 in all simulations. The cell size in all simulations is set to 4  ×  4  ×  5 nm^3^, which ensures good numerical accuracy and computational efficiency. The initial magnetic texture of the micromagnetic simulation is set to a conical state in accordance with the experimental results. Figure 1a,b shows the result of β  =  0.01 and *J*  =   ± 1.0 × 10^12^ A·m^−2^, respectively.

## Author Contributions

X.Z.Y. conceived the project, performed the experiments, analyzed the results, and wrote the manuscript. S.F. and X.Z. conducted micromagnetic simulations under the supervision of M.M. and F.K. N.K., K.K., Y.Taguchi., and Y.Tokura. prepared the FeGe and Co_8.5_Zn_8.5_Mn_3_ samples. Y.L.C. and K.N. fabricated the bulk samples into thin plates. All authors discussed the data and commented on the manuscript.

## Funding

This work was supported in part by Grants‐In‐Aid for Scientific Research (Grant Nos. 20H00337, 23H05431, 23H04522, 23H01841,25K17939, 25H00611, 24H02231) from the Japan Society for the Promotion of Science (JSPS), by the Japan Science and Technology Agency (JST) CREST program (Grant No. JPMJCR20T1), and by the RIKEN TRIP initiative (Many‐body Electron Systems and Advanced General Intelligence for Science Program). M.M. also acknowledges the support of the Waseda University Grant for Special Research Projects (Grant No. 2025C‐133).

## Conflicts of Interest

The authors declare no conflicts of interest.

## Supporting information




**Supporting File 1**: advs74932‐sup‐0001‐SuppMat.docx.

**Supporting File 2**: advs74932‐sup‐0002‐MovieS1.mp4.

**Supporting File 3**: advs74932‐sup‐0003‐MovieS2.mp4.

**Supporting File 4**: advs74932‐sup‐0004‐MovieS3.mp4.

**Supporting File 5**: advs74932‐sup‐0005‐MovieS4.mp4.

**Supporting File 6**: advs74932‐sup‐0006‐MovieS5.mp4.

**Supporting File 7**: advs74932‐sup‐0007‐MovieS6.mp4.

## Data Availability

The data that support the findings of this study are available on request from the corresponding author. The data are not publicly available due to privacy or ethical restrictions.
