# Supplementary material for: Current‐Driven Nonreciprocal Response of Nonequilibrium Skyrmions
Source: Adv Sci (Weinh). 2026 Mar 20;13(31):e24224. doi: 10.1002/advs.202524224 (PMC13587986; doi:10.1002/advs.202524224)
Supplement: Supplementary file 1 [file ADVS-13-e24224-s001.docx]

Supporting Information

**Current-Driven Nonreciprocal Response of Nonequilibrium Skyrmions**

*Xiuzhen Yu******, *Soju Furuta*, *Xichao Zhang*, *Yi-Ling Chiew*, *Kiyomi Nakajima*, *Kosuke Karube*, *Naoya Kanazawa*, *Yasujiro Taguchi*, *Yoshinori Tokura*, *Masahito Mochizuki*, *Fumitaka Kagawa*

**Skyrmion formations under uniform/nonuniform current excitations**

Compared to the uniform current flowing through chiral-lattice magnets like FeGe, a nonuniform electric current has a lower threshold for generating magnetic skyrmions in nontopological states, such as conical and ferromagnetic states, as shown in Fig. S1.

**Figure S1. Magnetic field dependence of the critical current density *J*_C_ required to create skyrmions in the conical and ferromagnetic states, with uniform (red curved line) and nonuniform (blue curved line) current flows in a chiral-lattice FeGe thin plate.** Insets show single skyrmion textures formed in the conical and ferromagnetic states, respectively.

**Magnetic phase diagrams of thin plates of FeGe and Co_0.5_Zn_0.5_Mn_3_**

Figure S2 shows the magnetic phase diagrams of thin FeGe (**a**) and Co_8.5_Zn_8.5_Mn_3_ (**b**) in the *T*-*B* plane, observed via *in situ* Lorentz TEM with varying *T* and *B* which results from changes in the objective lens current of the transmission electron microscope. Both phase diagrams indicate that the helical spin structure at lower bias fields and the conical spin textures at higher bias fields are thermodynamically stable over a wider temperature range—below 250 K for FeGe and below 300 K for Co_8.5_Zn_8.5_Mn_3_. In contrast, the skyrmion lattice is thermodynamically stable only within a narrow temperature range near the spin ordering transition temperature, approximately 278 K for FeGe^16^ and 340 K for Co_8.5_Zn_8.5_Mn_3_^40^.

**Figure S2: Magnetic phase diagrams of thin plates of FeGe (a) and Co_8.5_Zn_8.5_Mn_3_ (b), respectively, in the temperature *T*-normal magnetic bias *B* plane.** H, C, FM and SkL represent the helices with in-plane wavevectors, the conical spin textures with out-of-plane wavevectors, the ferromagnetic structure and a hexagonal skyrmion lattice state, respectively.

**Asymmetric dynamics of skyrmion formations under current reversals**

**Figure S3: Current crowding induces nonreciprocal dynamics of magnetic skyrmion formations in a chiral magnet Co_8.5_Zn_8.5_Mn_3_ thin plate with a hole of 200 nm diameter. a**-**h** Lorentz TEM images observed under a 50-mT perpendicular field at 250 K. **a**-**b** A Lorentz TEM image (**a**) and corresponding phase image (**b**) showing a mixture of a conical domain with an out-of-plane wavevector and in-plane helices, with the phase boundary marked by solid yellow lines. The inset displays the corresponding field map around the hole, illustrating curved spin textures guided by dashed yellow lines. **c**-**e**, **f**-**h** Snapshots taken at different elapsed times under current flow with *J* = 2.2×10^9^ A·m^−2^, and *J* = −2.2×10^9^ A·m^−2^.

A series of *in situ* Lorentz TEM images (Fig. S3) were captured under current flow. A Lorentz TEM image (**a**) and the corresponding phase image (**b**) depict the initial state of a mixture of in-plane helices and conical domains (monotone contrast) under a bias field of 50 mT at 250 K in the Co_8.5_Zn_8.5_Mn_3_ plate. After applying a 2.2×10^9^ A·m^−2^ current through the Co_8.5_Zn_8.5_Mn_3_ plate, a single skyrmion begins to form around the hole (**c**). The number of skyrmions increases with prolonged current flow time (**d**). Interestingly, skyrmions crystallize on the upper side of the hole (**e**) but gather on the lower side near the hole when the current flows in the opposite direction, while other parameters, such as *T* and *B*, maitained at *J* = -2.2×10^9^ A·m^−2^ (**f**-**h**).

**Modifications of magnetic states under electric current flow in the FeGe**

The thermodynamically stable magnetic states in the FeGe are shown in Fig. S4**a** and Fig. S4**c**, illustrating a helical state with a multidomain structure at zero field bias and a SkL state at a 55-mT bias, respectively. The multidomain structure transitions into a single domain, where the helices align along the current direction (Fig. S4**b**). In contrast, the SkL state (Fig. S4**c** and Fig. S4**e**) collapses around holes (Fig. S4**d** and Fig. S4**f**) when the current flows through the FeGe containing holes, demonstrating the detrimental effect of defects in erasing skyrmions.

**Figure S4: Modification of the wavevector of in-plane helices and collapse of thermally dynamic stable skyrmions around the hole in the FeGe plate with holes. a-b.** Lorentz TEM micrographs taken at 120 K: (**a**) without external stimuli (bias field or electric current) and (**b**) after applying a current of 2.8×10^9^A·m^−2^. **c-d.** Lorentz TEM images showing (**c**) a thermodynamically stable hexagonal SkL at 250 K, near the transition temperature, and (**d**) its collapse under a ⎯1.2×10^9^ A·m^−2^ current flow. **e-f.** Sketches traced from the Lorentz TEM images in (**c**) and (**d**), respectively, for easier visual reference. Red arrows indicate diffraction contrasts caused by the thickness gradient of the plate sample or local strains.

Figure S5: Nonreciprocal skyrmion responses under current *J* flow at different bias magnetic fields *B* and temperatures *T*. The symbols denote data points acquired through in situ Lorentz TEM measurements. The parameter $d$indicates the displacement of domain walls between skyrmions and non-topological spin textures.
